# Antimicrobial Activity of Submerged Cultures of Endophytic Fungi Isolated from Three Chilean *Nothofagus* Species

**DOI:** 10.3390/jof12010077

**Published:** 2026-01-21

**Authors:** Héctor Valenzuela, Daniella Aqueveque-Jara, Mauricio Sanz, Margarita Ocampo, Karem Henríquez-Aedo, Mario Aranda, Pedro Aqueveque

**Affiliations:** 1 Laboratory of Microbiology and Mycology Applied, Department of Agroindustries, Faculty of Agricultural Engineering, University of Concepcion, Chillan 3780000, Chile; hvalenzuela2016@udec.cl (H.V.); daqueveque2017@udec.cl (D.A.-J.); msanz2016@udec.cl (M.S.); mocampo@udec.cl (M.O.); 2 Laboratory of Biotechnology and Food Genetics, Basic Sciences Department, Faculty of Sciences, University of Bio-Bio, Chillan 3780000, Chile; kahenriquez@ubiobio.cl; 3 Laboratory of Food & Drug Research, Department of Pharmacy, Faculty of Chemistry and Pharmacy, Pontifical Catholic University of Chile, Santiago 8320000, Chile; mario.aranda@uc.cl

**Keywords:** endophytes, Chilean *Nothofagus* host, submerged fermentation, bioprospecting, antimicrobial activity

## Abstract

Endophyte fungi (EF) are considered a new and valuable reservoir of bioactive molecules of biotechnological interest for pharmacy, agricultural and forestry industries. In this study, thirty EFs, isolated from three Chilean *Nothofagus* species (*N. alpina*, *N. dombeyi*, *N. oblicua*) were identified and cultured in submerged liquid fermentations aimed at searching for natural active substances. The extracts obtained were evaluated against pathogenic bacteria and fungi. Sixteen extracts (53.3%) presented antibacterial and fourteen (46.6%) presented antifungal activities in different intensities. Extracts from isolates *Coryneum* sp.-72 and *P. cinnamomea*-78 exhibited the highest antimicrobial activity. Using bioautography, the compounds responsible for the antimicrobial activity exhibited by *Coryneum* sp.-72 and *P. cinnamomea*-78 were detected and characterized. *Coryneum* sp.-72 showed bactericidal properties at 200 μg/mL and bacteriostatic effects at 50 μg/mL against *B. cereus*, *B. subtilis*, *L. monocytogenes* and *S. aureus*. MIC values indicated that *P. cinnamomea*-78 exhibited a strong fungistatic and fungicidal effect against *B. cinerea* and *C. gloesporioides* at 10–50 μg/mL. Isolates were grouped in the following order: Botryosphaeriales, Diaporthales, Eurotiales, Helotiales, Hypocreales, Pleosporales, Magnaporthales, Sordariales and Polyporales. EF isolated, identified and evaluated constitute the first report for Chilean *Nothofagus* genus.

## 1. Introduction

Traditionally, plants and microorganisms have been of great relevance to the agrochemical and pharmaceutical industries, both as sources of new bioactive molecules and drugs and as a reservoir of chemical diversity for the investigation focused at discovering new drugs. The identification of novel bioactive natural products is an arduous and continuous process, and new sources are constantly being explored. In the context of exploring novel sources of biologically active compounds, pharmaceutical and agrochemical industries are now directing their attention towards groups of organisms that previously received insufficient attention. These organisms include diverse groups of bacteria, algae, marine organisms and fungi [1].

Fungi constitute a significant proportion of the global genetic diversity, and their diversification and evolution had important impacts for the development of life on Earth [2]. Currently, the total diversity of fungi has been estimated at 2.2–3.8 million species [3,4]. They are predominated by Ascomycetes, Basidiomycetes and Chytridiomycota in marine environments, while terrestrial environments are generally dominated by Ascomycetes and Basidiomycetes [5,6]. Among the fungi, the so-called endophytic fungi (EF) or mycoendophytes are of particular interest. By definition, an EF is an endosymbiont that lives in mycelial form within a plant for at least part of its life cycle without causing apparent disease [7]. According to the global documentation, a total of 2771 EF from 877 genera have been reported, including 2224 species [8]. This value constitutes less than 1% of the total estimated number of EF. In a recent study, Bhunjun et al. [9] calculated that there are over 3 million species of EF. EF are ubiquitous and have been detected in all species of plants studied to date. EF are known to confer host plant adaptation to stresses and promote growth through phytohormone production and nutrient acquisition [10]. Current research on EF is focalized at a better understanding of ecology, evolution, their impact on ecosystems as well as their inexhaustible reservoir of biologically active natural compounds [11,12,13,14]. Scientific reports indicate that EF accumulates a wealth of active and structurally diverse natural products that are unprecedented in nature and are very important for drug discovery or as lead compounds for agriculture, medicine and pharmacy [15,16,17]. They produce a wide range of substances of different chemical classes, including steroids, xanthones, phenols, isocoumarines, perylene derivatives, quinones, furandiones, terpenoids, depsipeptides and cytochalasine, polyketides, alkaloids, peptides, proteins, lipids, shikimates, glycosides and isoprenoids [18,19,20,21,22,23]. Some compounds have shown interesting pharmacological activities, including antimicrobial, antioxidant, anti-diabetic, anti-malarial, antitumor, insecticidal and herbicidal properties [24,25,26,27].

The detection of active compounds in crude extracts is a time-consuming and costly process. Thin-layer chromatography (TLC) is a commonly used technique for separating and identifying the components of a mixture. TLC-bioautography is a fast, effective, economical and widely accepted method for assessing the inhibitory effect of compounds present in natural extracts against bacteria, yeasts and filamentous fungi. Also, this technique has the capacity to classify metabolites into different types of molecules by utilizing a variety of specific reagents [28,29]. In the present study, the active substances produced by the isolated EF were detected using TLC-bioautography.

Chile’s expansive geographical territory encompasses a wide array of flora, influenced by its diverse climatic conditions. The Chilean flora exhibits a high degree of endemism compared to the flora of other countries in South America, with 4 endemic families and 83 endemic genera, encompassing approximately 2145 endemic species [30]. Undoubtedly, Chilean flora offers a huge reservoir to discover new EF species and identify potential producers of novel and bioactive compounds. Despite this enormous potential, the investigations oriented to the study of the Chilean EF are scarce and poorly documented. The present study describes the investigation on EF isolated from Chilean endemic plants (dicots) belonging to the genus *Nothofagus* as producers of active compounds. *Nothofagus*, also known as the southern beeches, is a genus of 43 species of shrubs and trees native to the Southern Hemisphere (Chile, Argentina, Australia, New Zealand, New Guinea, New Caledonia). The genus has a rich fossil record of leaves, cupules and pollen with fossils extending into the late Cretaceous period. Additionally, the genus *Nothofagus* has been central to reconstructions of the biogeography of Gondwana, plays a role in the ecological dynamics of forests where *Nothofagus* is present, and can host extremely diverse communities of vascular and non-vascular plants, epiphytes/endophytes, bacteria, yeast and fungi [31,32]. In Chile, all species belong to the subgenus *Nothofagus* and are present as part of the Andean–Patagonian forests. Taxa within subgenus *Nothofagus* are ancient lineages that have developed adaptations throughout their evolutionary history. However, pressures from exotic plantations, diseases, pests, fire, timber harvesting and climate change are the current and immediate threats that are changing the dynamics of *Nothofagus* forest ecology and affecting the future of these species [33]. These facts would indicate that the Chilean *Nothofagus* species are a source of EF with potential to produce natural and active substances. Thus, it is necessary to evaluate this biological and chemical wealth before human intervention in our forests causes their disappearance. Therefore, the main objective of this study is to carry out the first bioprospecting of EF from Chilean *Nothofagus* species and explore their capacity to produce antibacterial and antifungal substances and their potential to become model chemical structures for new antimicrobials capable of replacing current antibiotics or synthetic fungicides.

## 2. Materials and Methods

### 2.1. Collection of Host Plants

The samples of *N. alpina*, *N. dombeyi* and *N. oblicua* were collected from Termas Chillán, close to the Andes Mountains, Ñuble Region, Chile. Mature and healthy leaves and stems were selected randomly from the aerial plant. The samples were placed in zipper lock bags and stored for less than 24 h at 4–5 °C prior to the isolation of EF [33]. An herbarium specimen was deposited in triplicate in the Herbarium Collection of the Laboratory of Microbiology and Mycology Applied at the University of Concepción, Chillán. The voucher specimens are as follows: *N. alpina* (Voucher LMM-P N° 052-23); *N. dombeyi* (Voucher LMM-P N° 053-23); and *N. oblicua* (Voucher LMM-P N° 062-23).

### 2.2. Isolation of EF

EF were isolated using the superficial disinfection procedure. Briefly, leaves and stems were washed thoroughly in running tap water to eliminate any soil or debris. Then, they were sterilized with 70% ethanol (1 min), sodium hypochlorite solution 2% (3 min) and 70% ethanol (30 s). All samples were rinsed three times with sterile distilled water. Small pieces of leaves (5 × 5 mm) and stems (5 × 10 mm) were excised using a sterile blade and were placed on PDA medium containing 50-µg/mL penicillin and streptomycin. The plates were incubated at 23–25 °C (Incubator: Zhicheng, mod. ZFD-5090, Shanghai, China) for up to 8–10 days and the hyphal tip of each fungus growing out from the plant tissue was transferred to a new PDA plate [34]. Microscopic records of the EF were generated using a microscope (Bel-Photonics, mod. Bio-2T-PL, Monza, Italy) connected to a camera (Bel-Photonics, mod Eurekam 6-PLUS, Italy).

### 2.3. Identification of the Fungal Isolates

EF were identified either morphologically based on characteristics of the fungal culture (appearance of colony, structure and color of mycelium texture; spores: size, color, shape and hyphal diameters) or molecularly through analysis of the internal transcribed spacer regions of nuclear ribosomal DNA (ITS1-5.8S-ITS4 rDNA). Total DNA was isolated from eight-day-old fresh mycelia with a DNA extraction kit (E.Z.N.A^®^ Omega Bio-Tek Inc, Norcross, GA, USA) and subjected to PCR (Thermocycler, mod. Applied Biosystems™ MiniAmp™ Plus, Thermo Fisher Scientific Inc., Waltham, MA, USA) to amplify ITS1-5.8S-ITS4 rDNA region using the following universal primers, ITS1 (5-TCCGTAGGTGAACCTGCGG-3) and ITS4 (5-TCCTCCGCTTATTGATATGC-3) [6,7]. The ITS region was analyzed by mixing 1 µL DNA [20 ng µL^−1^]; 0.2 µL Taq polymerase Go Taq G2 (Promega, Fitchburg, WI, USA) [5 U µL^−1^]; 2 µL primer ITS1 (10 µM); 2 µL first ITS4 (10 µM); 0.8 µL dNTPs (10 mM); 8 µL buffer [5×] (buffer contain MgCl_2_ [7.5 mM]); and 26 µL ultrapure water. The thermocycler parameters were as follows: an initial cycle of 5 min at 94 °C; 35 cycles of 1 min at 94 °C, 30 s at 55.5 °C and 1 min at 72 °C; and a final cycle of 10 min at 72 °C. The PCR products were analyzed for gel electrophoresis using a 1% agarose gel dissolved in Tris-Acetate-EDTA buffer (1×), stained with SYBR Safe DNA Gel stain and observed under UV light (Transilluminator-UV, Daihan, mod. MUV-M20, Seoul, Republic of Korea). The purified PCR products were subjected to sequence determination by a specialized Chilean agency (Austral-Omics, Valdivia, Chile). Sequences alignments were used to identify the isolates of interest using the BLAST search tool provided by the National Center for Biotechnology Information. ITS sequences were compared with reference sequences from NCBI GenBank using BLASTn. Sequences were aligned with the ClustalW algorithm in MEGA software version 5.2, and phylogenetic trees were constructed using the neighbor-joining (NJ) method with the Tamura 3-parameter substitution model. Node support was evaluated by 500 bootstrap replicates [35].

### 2.4. Submerged Fermentation

Ten to fifteen fungal mycelium pieces (5 × 5 mm in diameter) of each isolate were removed under sterile conditions and inoculated into 1 L Erlenmeyer flasks with 500 mL of liquid YMG medium composed of dextrose, yeast extract and malt extract (4, 4, 10 g/L); initial pH 5.5. The flasks were incubated at 20–22 °C in an orbital shaker (N-Biotek mod. NB-101MT, Bucheon, Republic of Korea) with constant agitation at 120 rpm. When abundant mycelia were observed, glucose was emptied and the pH was approximately 7, the cultures were stopped [36]. Then, the mycelium was removed via filtration in a Büchner funnel. This mycelium was dried at 40–45 °C and extracted with methanol (MeOH). The broth (liquid phase) was extracted with ethyl acetate (EtOAc) in a 1:1 ratio using a separation funnel. The EtOAc and MeOH were concentrated in an evaporator rotatory (Buchi, mod B-100, New Castle, DE, USA), yielding the EtOAc extract and MeOH-extract, respectively. Then, all extracts were dissolved in MeOH at a concentration of 10 mg/mL and frozen (−18 °C) for the biological assays [37].

### 2.5. Evaluation of Antimicrobial Activity

The antimicrobial activity of the extracts (EtOAc, MeOH) was determined in the serial dilution assay or the plate diffusion assay. Sterile paper filter disks of 6 mm diameter (Whatman Nº3 Merck, Darmstadt, Germany) were impregnated with 200 µg of the different extracts. Bacterial and fungal cultures were diluted with sterile water to obtain a microbial suspension of 10^6^–10^8^ CFU/mL and 10^5^–10^7^ spore/mL, respectively. Petri plates containing 20 mL of culture medium with 2% (*w*/*v*) molten agar were inoculated with 200 μL of microbial suspensions and allowed to solidify in a series chamber. After solidification, the disks were placed on the inoculated culture medium [38]. The positive controls were streptomycin (streptomycin sulphate) and Rovral^®^ 50% WP (Iprodione-Dicarboximides) for bacteria and fungi, respectively. The plates were incubated at 37 °C (bacteria) and 20–22 °C (fungi) overnight and the inhibition halo around the disks was measured (mm) after 24–48 h. The bacterial strains tested were *Bacillus cereus* (LMM-876), *B. subtilis* (ATCC 6633), *Enterobacter aerogenes* (LMM-141), *Escherichia* coli (ATCC 259522), *Klebsiella pneumoniae* (ATCC 13883), *Listeria monocytogenes* (LMM-105), *Salmonella entiritidis* (LMM-352), *Staphylococcus aureus* (ATCC 25923), *S. epidermidis* (LMM-322) and *S. aureus*-MRSA (ATCC 33591). The fungal strains used were *Alternaria alternata* (LMM-010), *Aspergillus niger* (LMM-110), *Botrytis cinerea* (LMM-097), *Cladosporium cladosporioides* (LMM-093), *Fusarium oxysporum* (LMM-034), *Penicillium expansum* (LMM-012) and *Rhizopus stolonifer* (LMM-047). All extracts were tested in duplicate. The extracts exhibiting the highest antimicrobial activity were considered to possess potential antimicrobial activity and selected to determine the inhibition of mycelial growth, inhibition of sporogenesis and determine the minimal inhibition concentration.

#### 2.5.1. Mycelial Growth Inhibition (MGI%)

Extracts that evidenced positive and potent activity against fungi tested were selected for the determination of the MGI. EtOAc extracts were diluted at different concentrations (10, 50, 100, 200, 500, 1000 and 2000 µg/mL). The diluted samples were deposited and uniformly distributed on the surface of Petri dishes with PDA media. The same proportion of ethanol and Rovral^®^ 50% WP was added on untreated plates. Unmodified plates were used as controls. Mycelial disks of 5 mm in diameter obtained from the periphery of 8–10-day-old cultures of the *A. alternata*, *A. niger*, *B. cinerea* and *C. gloesporioides* were placed upside down on the center of Petri dishes. This was performed for each different extract and concentration. All dishes were incubated at 20–22 °C in darkness. Each extract was tested in triplicate. Three radiuses of the mycelium were measured each day until the mycelium reached the edge of the plate. MGI was calculated as follows: MGI% = [(DC − DO)/DC] × 100, where DC is the average of colonies diameter in untreated dishes and DO is the average of colonies diameter in dishes treated with different concentrations of EtOAc extracts [39].

#### 2.5.2. Spore Germination Inhibition (SGI%)

To evaluate the activity of the EtOAc extracts on spore germination, the microcultures method (96-wells) was employed. Diluted concentrations of active EtOAc extracts were placed in the wells (10, 50, 100, 200, 500, 1000 and 2000 µg/mL), and 200 µL of the spore suspension (10^5^–10^6^ spore/mL) was deposited into each well. Rovral^®^ was utilized as the positive control while conidial suspensions were employed as the control negative in the untreated wells. The microdilution plates were maintained at a temperature of 20–22 °C for a period of 24 h. Thereafter, 100 spores from each assay were observed under microscope (40×) to establish the number of germinated spores. Spores were considered germinated if they produced germ tubes at least twice their width. SGI was calculated as follows: SGI (%) = 100 (A − B)/A, where A is the percentage of germinated spores in control and B is the percentage of germinated spores in the sample. The experiments were in triplicate [40].

#### 2.5.3. Minimal Inhibitory Concentration (MIC)

The most active extracts with positive activity against one or more of the bacteria or fungi were selected to evaluate the MIC. Ninety-six well microdilution plates were used. Briefly, different concentrations of the active extracts (10, 50, 100, 200, 500 µg) were deposited in the wells before the microorganism-test (200 μL) was added. The positive control was penicillin G and streptomycin (streptomycin sulphate) and Rovral® 50% WP (Iprodione-Dicarboximides) for bacteria and fungi, respectively. The positive control was penicillin G and streptomycin (streptomycin sulphate) and Rovral® 50% WP (Iprodione-Dicarboximides) for bacteria and fungi, respectively. Each extract was tested in triplicate and the plates incubated for 24–48 h. The turbidity observed in the wells was interpreted as indicative of visible growth of the test microorganisms. The lowest concentration at which no turbidity was observed after incubation was considered the MIC value of the extract, indicating its bacteriostatic or fungistatic effects. The contents of the wells (10 μL) resulting from MIC were streaked using a sterile wire loop on Petri dishes with Müller-Hinton/PDA medium and incubated at 37 °C or 20–22 °C for 24–48 h. The lowest concentrations of the extracts that exhibited no bacterial or fungal development were considered as the MBC/MFC (minimal bactericidal/fungicidal concentration) [41].

#### 2.5.4. Thin-Layer Chromatography and Bioautography

To detect the active compound (s) with bactericidal/fungicidal activity produced by the isolates, thin-layer chromatography (TLC) coupled with bioautographic was carried out. Ten milligrams of EtOAc extract were dissolved in one milliliter of methanol to obtain 10 mg/mL of stock solutions. Stock solutions were then filtered, and 5 to 10 μL of each extract solution was spotted onto TLC silica gel plates (TLC Silica gel 60 F254; Merck, Santiago, Chile), with the help of a glass capillary tube.

The TLC plates were developed using chloroform/methanol (9:1) as eluent. After the solvent ran up to 90% of total height, the plate was air-dried to remove solvents and spots were visualized under UV light at 254 and 365 nm. Retention factor (Rf) values for the different spots were measured and calculated using the following formula: Rf = DSU/DSV, where RF is retention factor, DSU is the distance traveled by the solute (spot) and DSV is the distance traveled by the solvent.

For the bioautographic test, Petri plates with organism-test were prepared as indicated in Section 2.4. The eluted TLC plates were placed on the agar with the silica side down. The Petri plates were incubated at 37 °C (bacteria) and 20–22 °C (fungi) for 24–48 h. After the incubation time, halos of inhibition around of spots were observed and recorded [42].

All the chemicals and solvents used in the experiments were analytical grade with ≥95% purity and were obtained from E. Merck, Santiago, Chile.

### 2.6. Statistical Analysis

All the measurements were replicated in three independent experiments. Analysis of variance (ANOVA) of the mean values of MGI and IS was carried out with the statistical program STATGRAPHICS Centurion. Tukey’s HSD test was applied when ANOVA revealed significant differences (*p* < 0.05) [43].

## 3. Results

### 3.1. Identification of EF

All samples of *Nothofagus* were collected during summer 2023 in central Andean Mountains, Ñuble Region, Chile. 

**Figure 1:**
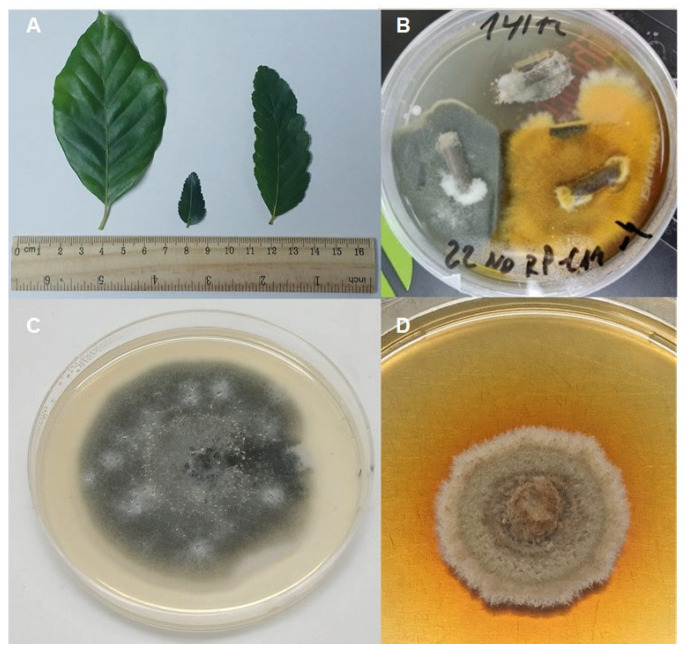
(**A**) Morphology of the leaves of the studied *Nothophagus* species (from left to right: *N. alpina*, *N. dombeyi*, *N. oblicua*). (**B**) Development of different EF from stems of *N. alpina*. (**C**) *Coryneum* sp.-72 and (**D**) *P. cinnamomea*-78.

The identification of the isolates was based on both morphological characteristics, as illustrated in Figures S1 and S2, and molecular analysis, as presented in Figures S3–S6. Preliminary identification based on ITS sequences using NCBI BLASTn tool is shown in Table 1. Thirty isolates were identified, four from *N. alpina*, twenty from *N. dombeyi* and six from *N. oblicua* (Figure 1). All endophyte taxa were placed in eight orders of Ascomycota (Botryosphaeriales, Diaporthales, Eurotiales, Helotiales, Hypocreales, Pleosporales, Magnaporthales and Sordariales) and one order of Basidiomycota (Polyporales). The analysis revealed that 96.6% of the EF belonged to Ascomycota, while the remaining 3.3% belonged to Basidiomycota. Based on the number of species, the orders with the most representatives were Diaporthales and Hypocreales with eight and six species, respectively. Additionally, the orders Helotiales and Pleosporales exhibited four species each, while the orders Eurotiales presented three species, Botryosporiales two species and Magnaporthales, Polyporales and Sordariales one species each. It was observed that orders Diaporthales and Hypocreales were the only ones that exhibited representatives isolated in the three plants studied. Fifteen genera were identified from the nine orders: Hypocreales (*Clonostachys*, *Fusarium Trichoderma*)*;* Pleosporales (*Alternaria*, *Paraconiothyrium*, *Stemphylium*); Helotiales (*Pezicula*, *Hymenotorrendiella*); Diaporthales (*Coryneum*, *Diaporthe*); Eurotiales (*Penicillium*); Botryosphaeriales (*Neofosicoccum*); Magnaporthales (*Neocucurbitarea*); Polyporales (*Phanerodontia*) and Sordariales (*Chaetomiun*). Only four isolates were identified in more than one host: *Clonostachys rosea*, *Diaporthe patagonica*, *Neofosicoccum nonquaesitum* from *N. dombeyi* and *N. oblicua*, and *Pezicula cinnamomea* from *N. alpina* and *N. dombeyi*.

### 3.2. Antimicrobial Activity of EF Extracts

Of the EtOAc extracts assayed, sixteen (53.3%) presented antibacterial and fourteen (46.6%) presented antifungal activities in different intensities against at least one of the tested microorganisms. Only three MeOH-extracts presented weak antimicrobial activity and thus were not considered interesting.

Of total extracts tested, the highest proportion of antibacterial activity occurred in members of the Hypocreales with six active extracts (20%), followed by order Diaporthales with four active strains (13.3%) and orders Helotiales and Pleosporales with two active isolate (6.6%) each. The orders Eurotiales and Sordariales each exhibited a single active isolate (3.3%). No antibacterial activities were observed in the orders Botryosporiales, Magnaporthales and Polyporales (Table 2).

In terms of antifungal activity, four isolates (13.3%) in the Diaporthales order were active against tested fungi. The orders Hypocreales, Helotiales, Eurotiales and Botryosphaeriales showed two isolated (6.6%) with antifungal activity each. The orders Pleosporales and Magnaporthales exhibited one isolate (3.3%) active each. No antifungal properties were observed in the orders Polyporales and Sordariales (Table 3).

**Figure 2:**
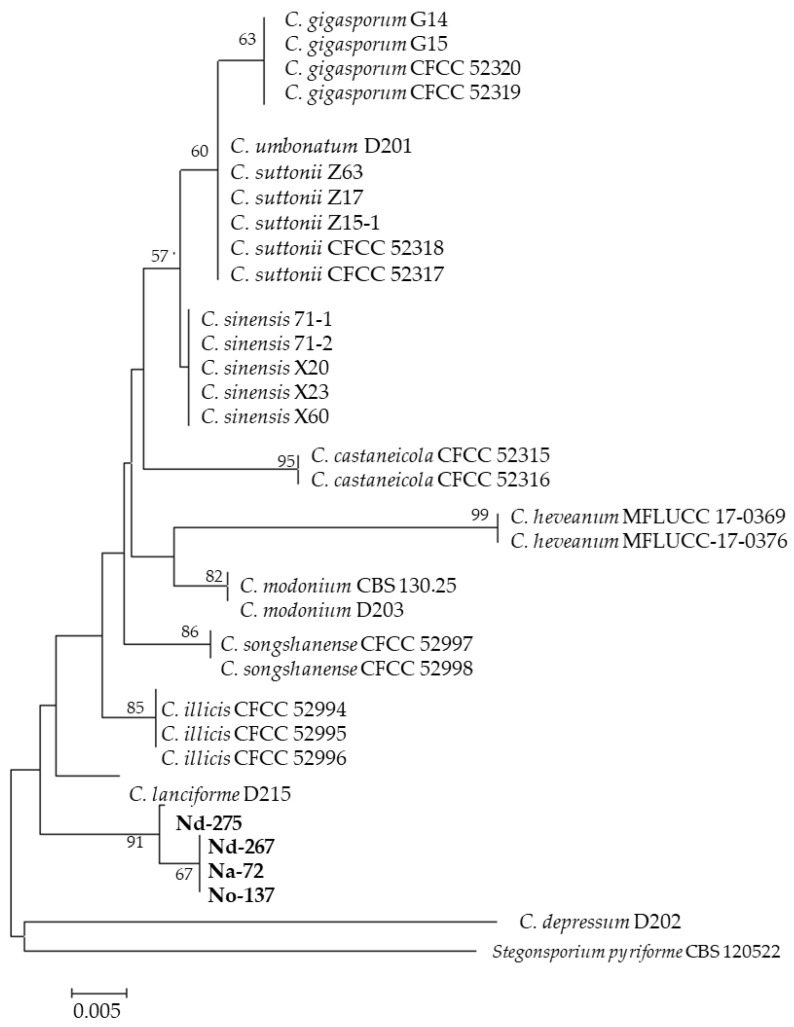
*Coryneum*. Neighbor-joining tree based on ITS sequences of isolates Na-72, No-137, Nd-267 and Nd-275 isolated from *Nothofagus* genus. The tree was rooted in *Stegonsporium pyriforme* (outgroup). Numbers labeled at each node indicate bootstrap value (%) from 500 replicates.

**Figure 3:**
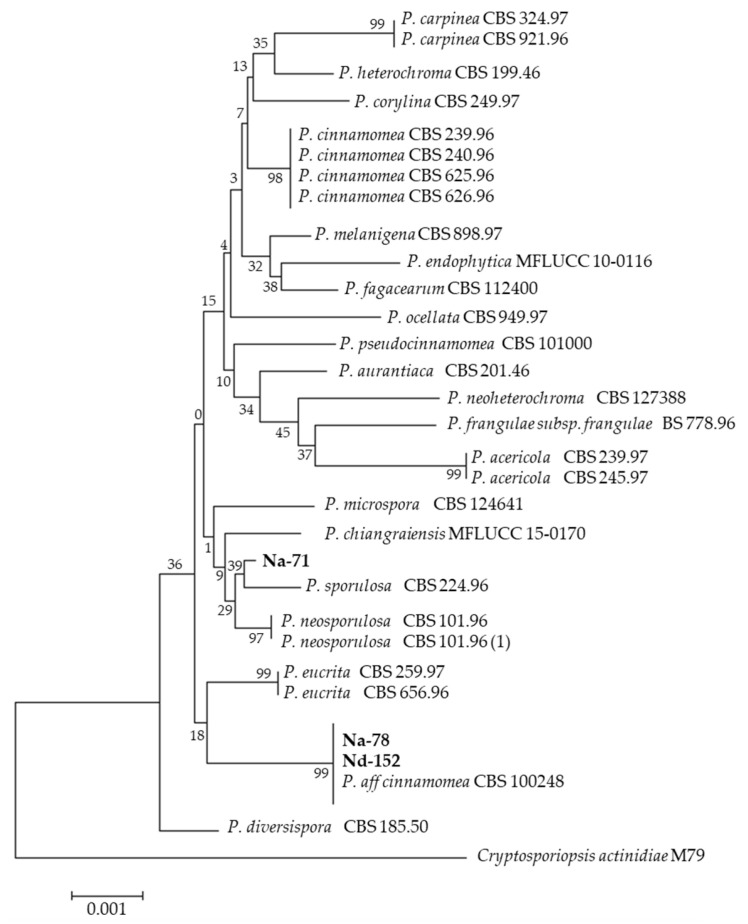
*Pezicula*. Neighbor-joining tree based on ITS sequences of isolates Na-71, Na-78 and Nd-152 isolated from *Nothofagus* genus. The tree was rooted in *Cryptosporiopsis actinidiae* (outgroup). Numbers labeled at each node indicate bootstrap value (%) from 500 replicates.

In this study, extracts that exhibited at least one inhibition zone larger than 15 mm in diameter were considered potentially promising and selected for further testing. Phylogenetic trees were constructed for the isolates that showed the highest level of antimicrobial activity. In this sense, only eight strains exhibited significant antibacterial activity. In the order Helotiales, the distinguishing isolates were identified as *F. petroliphilum* (Na-70), *F. tricinctum* (Nd-250), *Trichoderma* sp. (Nd-5) and *T. longibrachiatum* (Nd-5). In the orders Helotiales, Diaporthales and Pleosporales, they were *Coryneum* sp. (Na-72) (Figure 2), *P. cinnamomea* (Na-78) (Figure 3) and *Stemphylum* sp. (No-129), respectively. All these EtOAc extracts were able to inhibit the growth of Gram-positive (*B. cereus*, *B. subtilis*, *L. monocytogenes*, *S. aureus*, *S. epidermidis* and *S. aureus* MRSA) and Gram-negative (*E. aerogenes*) considerably.

For the antifungal activities, the orders Hypocreales (*C. rosea* (No-101)), Helotiales (*Pezicula* sp. (Na-71), *P. cinnamomea*-78), Diaporthales (*Coryneum* sp.-72) and Pleosporales (*Alternaria* sp. (Nd-258)) exhibited a strong effect against *A. alternata*, *A. niger*, *B. cinerea*, *C. gloesporioides*, *F. oxysporum*, *P. expansum* and *R. stolonifer*. In summary, the isolates *Coryneum* sp.-72, *P. cinnamomea*-78, *C. rosea*-101 and *Alternaria* sp.-258 were active against bacteria and/or fungi.

### 3.3. Minimal Inhibitory Concentrations

The MIC values for antibacterial evaluation are shown in Table 4. These indicate that *Coryneum* sp-72 exhibited bactericidal properties (MBC) at 200 μg/mL and bacteriostatic effects at 50 μg/mL against *B. cereus*, *B. subtilis*, *L. monocytogenes* and *S. aureus*. The strain *F. tricinctum*-250 showed bacteriostatic/bactericidal effects at 50/100 μg/mL toward *E. aerogenes* and *L. monocytogenes*. Also, *P. cinnamomea*-78 exhibited bacteriostatic/bactericidal effects at 50/100 μg/mL against *B. cereus*, *B. subtilis* and *S. aureus* while *C. rosea*-101 and *Stemphylum* sp.-129 showed bacteriostatic/bactericidal activities at 200/500 μg/mL toward *S. aureus*. At the highest concentration of extracts tested (500 μg/mL) some showed only bacteriostatic effects such as *F. petroliphilum*-70, *P. cinnamomea*-78, *Trichoderma* sp.-5 and *T. longibrachiatum*-9.

As demonstrated in Table 5, the MIC values indicate that *P. cinnamomea*-78 exhibited a strong fungistatic and fungicidal (MFC) effect against *B. cinerea* and C. *gloesporioides* at 10–50 μg/mL. However, the same strain exhibited higher values for the fungistatic/fungicidal control of *A. alternata* and *A. niger* (100–500 μg/mL) and 200/500 μg/mL for *F. oxysporum*, *P. expansum* and *R. stolonifer*.

Furthermore, *Pezicula* sp.-71 and *Coryneum* sp.-72 exhibited fungistatic/fungicidal effect towards *A. alternata*, *A. niger* and *C. gloesporioides* at 100–500 μg/mL. Both strains showed a fungistatic control against *R. stolonifer* at 500 μg/mL. The isolate *C. rosea*-101 showed fungistatic/fungicidal effect toward *A. alternata*, *P. expansum* and *R. stolonifer* at 100/500 μg/mL, respectively. Also, *C. rosea*-101 exhibited a fungistatic effect against *C. gloesporioides* at 500 μg/mL. A similar range of values (100–500 μg/mL) were observed by *Alternaria* sp.-258 against *A. niger* and *C. gloesporioides*.

The MIC values indicate that the EtOAc extracts were more effective in inhibiting fungal than bacterial growth at lower concentrations. Moreover, the results obtained demonstrate that the strains isolated from *N. alpina*, including *F. petroliphilum*-70, *Pezicula* sp.-71, *P. cinnamomea*-78 and *Coryneum* sp.-72, are noteworthy for their interesting antifungal property, especially *P. cinnamomea*-78 that showed the lowest concentrations to inhibit or control the tested fungi.

### 3.4. Mycelial Growth Inhibition (MGI) and Spore Germination Inhibition (SGI)

MIC assay data indicated that *P. cinnamomea*-78 and *Coryneum* sp.-72 exhibited higher and moderate antifungal activity, respectively. Consequently, both were selected for further study in the MGI and SGI tests. The results of the different concentrations of EtOAc extracts produced by *P. cinnamomea*-78 on the mycelial growth and spore germination are shown in Table 6. This extract was able to inhibit the mycelial growth of four fungi tested at varying levels of intensity. At concentrations of 1000 and 2000 µg/mL, it was significantly active, inhibiting mycelial growth of *B. cinerea* by 60% and 77% and that of *C. gloesporioides* by 57% and 64%, respectively. Nevertheless, this extract was not significantly active against *A. alternata* (34, 42%-MGI) and *A. niger* (32, 49%-MGI), reaching lower values of inhibition at the same concentrations. At concentrations of 10 to 500 µg/mL, the extract of *P. cinnamomea*-78 demostrated moderate activity toward *A. alternata*, *A. niger*, *B. cinerea* and *C. gloesporioides*. Statistical analysis revealed that the MGI index varied when different concentrations of the extract were utilized at the range of 1000 and 2000 µg/mL. However, no significant difference was observed at the range of 10 to 500 µg/mL. The results established that the *P. cinnamomea*-78 extract-EtOAc was more effective in inhibiting spore germination of *B. cinerea* and *C. gloesporioides* than other fungi tested (*p* = 0.05). At 10 µg/mL, the germination was inhibited in 52% and 54%, respectively, and reaching 100% from 500 µg/mL. Spore germination of *A. alternata* and *A. niger* was inhibited by nearly 90% at 2000 µg/mL.

The effects of EtOAc extract of *Coryneum* sp.-72 on mycelial growth and spore germination are shown in Table 7. At highest concentrations evaluated (1000 and 2000 µg/mL), the extract was not able to inhibit the mycelial growth of tested fungi significantly, reaching 45% (*A. alternata*), 33% (*A. niger*), 43% (C. *gloesporioides*) and 25% (*R. stolonifer*) as MGI index. At concentration of 100 µg/mL, the extract inhibited the spore germination of *A. alternata*, *A. niger, C. gloesporioides* and *R. stolonifer* in 58%, 54%, 52% and 12% as SGI index, respectively. The extract presented an index close to 90% at 2000 µg/mL for *A. alternata*, *A. niger* and *C. gloesporioides*. Furthermore, it is evident that, as the concentration of the extract increased, the SGI also decreased. In the absence of extract, no mycelial growth and spore germination inhibition was detected.

### 3.5. Detection of Bioactive Compound(s) Using TLC-Bioautography

**Figure 4:**
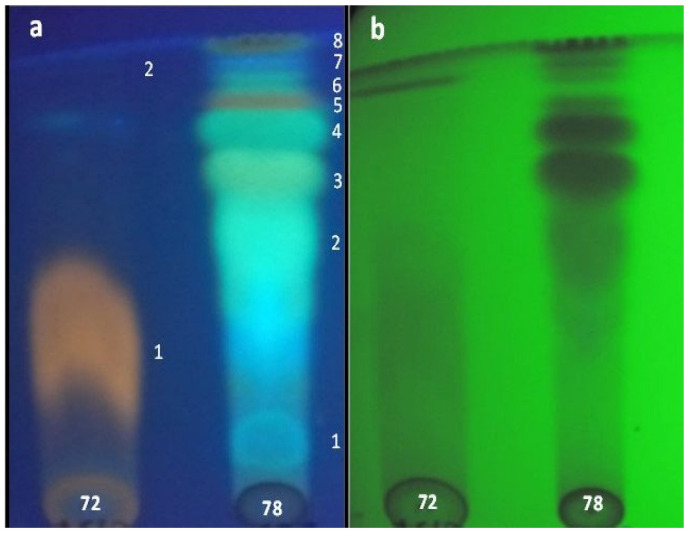
TLC development in ChCl_3_: MeOH (9:1) of EtOAc extracts from *Coryneum* sp.-72 and *P. cinnamomea*-78 visualized under UV light at 365 (**a**) and 254 nm (**b**). Numbers and colors represents individual spots.

TLC analysis revealed that *Coryneum* sp.-72 and *P. cinnamomea*-78 exhibited two and eight spots (compounds), respectively, with distinct *Rf* values and appearance under UV-light (Table 8). The bioautography of *Coryneum* sp.-72 (Figure 4) showed that only the designated compound **1** exhibited a well-detectable halo of inhibition against *S. aureus*. This effect was similar toward *B. cereus* and *B. subtilis* and weakly detectable against *L. monocytogenes*, *A. alternata*, *A. niger* and *C. gloesporioides*. Compound **2** was not active against the bacteria and fungi evaluated.

**Figure 5:**
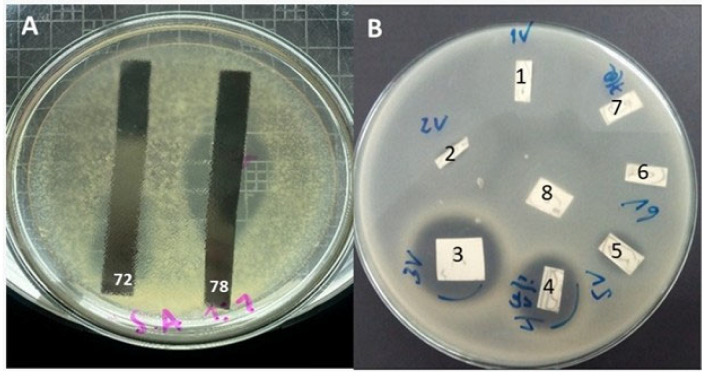
(**A**) Bioautography assay of EtOAc extracts from *Coryneum* sp.-72 and *P. cinnamomea*-78 on *S. aureus*. (**B**) Bioautography of separated spots (compounds) of *P. cinnamomea*-78 on *S. aureus*.

In the case of *P. cinnamomea*-78, bioautographic assay revealed the presence of a unique halo of inhibition positioned centrally on the TLC plate. As demonstrated in Figure 4, the halo surrounds compounds **3**, **4** and **5**, and it remains uncertain whether one or more of them are responsible for the observed inhibitory effect (Figure 5A). To elucidate these results, a modification of the bioautography technique was employed, whereby each individual spot was meticulously excised and then tested separately against the most sensitive test-organisms. Following the incubation period, the results indicated that compounds **3**, **4** and **5** independently exhibited antimicrobial effects, each forming its own zone of inhibition with different intensities and against different test organisms (Figure 5B). Compound **3** showed a very well-detectable halo of inhibition against *B. cereus*, *B. subtilis*, *S. aureus*, *B. cinerea* and *C. gloesporioides*. Additionally, this compound produced a well detectable halo against *A. alternata* and *A. niger*. A weak detectable halo was observed on *L. monocytogenes*, *S. epidermidis*, *S. aureus* MRSA, *F. oxysporum*, *P. expansum* and *R. stolonifer*.

Compound **4** produced a very well-detectable halo only towards *S. aureus*. It also exhibited a well-detectable halo against *B. cereus*, *B. subtilis*, *A. alternata* and *C. gloesporioides*. By contrast, the halo was weakly detectable against *S. epidermidis* and *S. aureus* MRSA, *A. niger* and *B. cinerea*. In the case of compound **5**, bioautography revealed that the halo of inhibition was only weakly detectable against *B. cereus*, *B. subtilis* and *S. aureus*, and negative on the other organisms evaluated.

## 4. Discussion

To date, the range of functions of EF and their interactions with the host and with other organisms associated with plants remain unclear. However, scientific evidence has revealed a high richness of EF species, impacting the taxonomy, systematic, mycological diversity, ecology and distribution of this group of organisms. Scientific information has revealed that EF directly influences plant metabolism, facilitating resistance to extreme temperatures, periods of drought and the presence of phytopathogens. This microbial biodiversity together with the chemodiversity of secondary metabolites produced by EF offer the opportunity for the search and discovery of new bioactive and natural compounds with potential uses in medicine, pharmacy and agronomy [44,45].

Research Chile has focused predominantly on the diversity of fungal communities and the ecological functionality of EF associated with plants collected from the Chilean desert and Antarctica, which are subjected to conditions of drought and heat stress. Some of these EF (extremophiles) have been inoculated into crops highly susceptible to conditions of stress, such as blueberries, tomatoes, lettuce and strawberries, allowing them to tolerate environmental stresses and emerging as a new and novel strategy to improve crop performance under climate change [46,47,48,49,50,51,52,53,54].

Conversely, research endeavors directed towards the exploration of Chilean EF as a potential reservoir of bioactive compounds are limited and remain underdeveloped. In this context, Schmeda-Hirschmann et al. [55] and Hormazabal et al. [56] published the first reports on EF isolated from Chilean gymnosperms. Over thirty-eight isolates were cultured in vitro and the extracts produced were evaluated against pathogenic bacteria and fungi. Some active compounds were identified, including 4-(2-hydroxyethyl) phenol, p-hydroxybenzaldehyde, mullein, peniprequinolone and gliovictin.

Furthermore, EF isolated from the Chilean tree *Embothrium coccineum* were reported with weak antifungal activity against *B. cinerea* [57]. Then, Vidal et al. [58] reported the isolation of EF from seven native and endemic plants growing in Central Andean region in the Metropolitan Region of Chile. Three isolates showed antifungal activity against *B. cinerea*. The isolates were identified as two species of *Alternaria* and *Aureobasidium*. Later, Castro et al. [59] isolated ten EF from *Echinopsis chiloensis* (Cactaceae) and *Baccharis linearis* (Asteraceae). Two isolates inhibited the mycelial growth of *B. cinerea* by antibiosis and were identified as *Epicoccum* sp. and Pleosporales sp. Active compounds in the Pleosporales sp. extracts were identified as saponins and/or terpenes; meanwhile, in the *Epicoccum* sp. extracts, alkaloids and phenolic compounds were responsible for the antifungal activity.

In this work, EF isolated from three species of *Nothofagus* genus (*N. alpina*, *N. dombeyi* and *N. oblicua*) were cultured in vitro and evaluated as potential producers of active compounds. The genus *Nothofagus* has 35 species distributed in New Guinea, New Caledonia, Australia, New Zealand, Argentina and Chile, with 4 monophyletic subgenera: *Nothofagus*, *Fuscospora*, *Lophozonia* and *Brassospora* [60,61]. The subgenus *Nothofagus* is endemic to South America (Argentina and Chile). Most of the native Chilean forests are constituted by *Nothofagus* species, distributed predominantly in the Andes Mountains with 11 taxa, corresponding to 9 species and constituting one of the main natural resources of the country. *N. alpina*, *N. dombeyi* and *N. oblicua* are woody plants that can reach up to 45 m in height with an average lifespan of more than 500 years and capable of living at temperatures between 0 °C and 35 °C [62,63]. The studied species are found in extensive forests in Central Andean Mountains of Chile, which are dominated by *N. dombeyi* and *N. oblicua* and *N. alpina* in less abundance. The above-mentioned characteristics indicated that these species are interesting candidates for the exploration of the biodiversity of EF, as well as their potential as producers of bioactive natural products.

However, studies on EF isolated from *Nothofagus* are even more scarce. In this regard, the only report was published by Johnston et al. [64] who studied EF biodiversity patterns in *Nothofagus* species present in New Zealand’s forests (*N. menziesii*, *N. solandri*, *N. fusca*), identifying a total of 3258 isolates representing 54 morphotype groups. The isolates were restricted to the orders Dothideomycetes, Eurotiomycetes, Leotiomycetes, Sordariomycetes and Xillariales.

The present study is the first record of EF isolated from Chilean species of *Nothofagus*, with the objective of identifying microorganisms capable of producing active substances. Herein, we reported the identification of thirty EF isolated from shoots and leaves of *N. alpina*, *N. dombeyi* and *N. oblicua*. A total of fifteen genera were identified, of which *Coryneum* and *Diaporthe* exhibited four isolates each, *Penicillium* and *Pezicula* with three isolates, and *Alternaria*, *Clonostachys*, *Fusarium*, *Neofusicoccum* and *Trichoderma* showed two isolates each. All other genera were represented by one isolate each.

Many of these genera have often been reported either as saprobes, pathogens or as non-pathogen endophytes from wide range of hosts [65,66,67,68]. In Chile, some species have mainly been reported from fruit trees [69,70]. However, Perez et al. [71] reported for the first time the presence of *N. nonquaesitum* on *Araucaria araucana*, an endemic Chilean gymnosperm. Later, Zapata et al. [72] published three new species of *Diaporthe* on endemic Chilean plants: *D. araucanorum* on *A. araucana* (Araucariaceae), *D. foikelawen* on *Drimys winteri* (Winteraceae) and *D. patagonica* on *Aristotelia chilensis* (Elaeocarpaceae).

In our work, the isolates belonging to the genus *Coryneum* (code 72, 137, 275) were found in the stems of the three species studied and in the leaves (code 267) of *N. dombeyi*. For *Diaporthe*, all isolates were isolated from stems, three species were detected in *N. dombeyi* (*D. rudis*-150, *D. araucanorum*-269, *D. araucanorum*-274) and one from *N. oblicua* (*D. cynaroidis*-33). In the case of *Pezicula*, the three isolates were obtained exclusively from *N. alpina*, *Pezicula* sp.-71 from stems and *P. cinnamomea* (code 78, 152) from stems and leaves. For *Penicillium*, three species were isolated from *N. dombeyi*, *P. chrysogenum*-106 (stems) and *P. crustosum* (259, 276) from leaves and stems, respectively. Also, *N. nonquaesitum* was identified on leaves of *N. dombeyi* and *N. oblicua*. All these identified genera and species represent the first records of EF for *Nothofagus* in Chile. It is also observed that some of the EF were found inhabiting more than one plant studied, suggesting a probable common colonization pattern. Undoubtedly, these new records corroborate the enormous biodiversity of EF harbored by Chile’s endemic flora, as well as their impact on generating new knowledge about EF.

The evidence has demonstrated that EF can produce growth substances utilized by the host, secondary metabolites with antibacterial, antifungal and insecticidal properties and their mycelia can colonize the habitats of certain pathogenic fungi. These characteristics have stimulated the investigation of EF as biological control agents against a variety of tree diseases and as precursors of biologically active and structurally diverse natural compounds [73,74].

As mentioned above, species of *Coryneum* and *Pezicula* have been predominantly isolated from a variety of hosts inhabiting temperate regions of the world. Previous research conducted into the search for natural compounds from *Coryneum* and *Pezicula* are limited. However, some bioactive secondary metabolites have been identified, such as corynecandin (antifungal) from *C. modonium* and mycorrhizin A (antifungal/cytotoxic), isocoumarin derivatives (cis-4-hydroxymellein (antifungal), trans-4-hydroxymellein (antifungal), (3R,4S)-3,4-dihydro-4,5,8-trihydroxy-3-methylisocoumarin (antifungal)), mellein (antifungal, insecticidal, phytotoxic, cytotoxic), ramulosin (antifungal), butanedioic acid, cryptosporioptides A–C (antibiofilm), discosiolide (antibacterial/antifungal), peziculastatin (antibiofilm), ethisolide (antibacterial/antifungal/algicidal/herbicidal) and equisetin (antibacterial/antifungal/herbicidal) from *Pezicula* [75,76,77,78]. The above information will provide guidance on the chemical groups and types of molecules that could be responsible for the biological activities observed in the evaluated extracts.

This study showed that the isolates *Coryneum* sp.-72 and *P. cinnamomea*-78 were able to produce extracts with the highest antibacterial and antifungal capacity, as evidenced by the screening results. Bioautographic analysis of these extracts indicated that compound **1** in *Coryneum* sp.-72 and compounds **3**, **4** and **5** in *P. cinnamomea*-78 would be responsible for the antimicrobial activity exhibited. Though *P. cinnamomea* has been reported on more than 30 broad-leaved trees and more than 10 species of conifers [79], there is a lack of scientific evidence oriented towards the search for active molecules. Probably, the present study and antimicrobial properties demonstrated by *P. cinnamomea*-78 constitute the first report and its possible potential.

## 5. Conclusions

This study provides information on the EF isolated from three Chilean *Nothofagus* species and the capacity of producing extracts with antimicrobial effects when they are cultivated in vitro conditions.

This is the first report on the bioactivity of the EF that inhabit the aerial parts of these native plants of Chile. The information obtained showed that of the thirty extracts evaluated for antibacterial and antimicrobial potential, two of them, *Coryneum* sp.-72 and *P. cinnamomea*-78, exhibited promising antimicrobial effects against Gram-positive and Gram-negative bacteria, as well as against fungi. Additionally, isolates such as *Alternaria* sp.-258, *C. rosea*-101, *F. petroliphilum*-70, *F. tricinctum*-250, *Pezicula* sp.-71, *Trichoderma* sp.-5 and *T. longibrachiatum*-9 also generated extracts with promising activities. The identified isolates are new records of host associations from *Nothofagus* and Chile. These findings demonstrate that EF provide additional insight into Chile’s chemical and biological diversity, which remains largely unexplored. The discoveries presented here are of particular pertinence in the context of the ongoing decline in biodiversity, which is likely to result in the loss of undiscovered natural products that may prove to be of significant utility to humanity. This study provides an additional incentive for the exploration and conservation of biodiversity in our temperate forests.

## Figures and Tables

**Table 1:** Preliminary identification of fungal isolates based on BLASTn of the ITS region.

Isolate	Order	Species	Organ Isolation	Accession No.	Identity (%)
Na-70	Hypocreales	*Fusarium petroliphilum*	stem	JN235238	100
Na-71	Helotiales	*Pezicula* sp.	stem	JN198438	100
Na-72	Diaporthales	*Coryneum ilicis*	stem	NR171088	93.52
Na-78	Helotiales	*Pezicula cinnamomea*	leaf	MH857079	100
Nd-5	Hypocreales	*Trichoderma* sp.	stem	MK871186	100
Nd-9	Hypocreales	*Trichoderma longibrachiatum*	stem	MW756930	99.44
Nd-34	Hypocreales	*Clonostachys rosea*	stem	OQ891611	100
Nd-106	Eurotiales	*Penicillium chrysogenum*	stem	MF925705	100
Nd-150	Diaporthales	*Diaporthe rudis*	stem	MT256131	99
Nd-151	Pleosporales	*Alternaria alternata*	stem	MF061753	99
Nd-152	Helotiales	*Pezicula neocinnamomea*	stem	KR859218	100
Nd-250	Hypocreales	*Fusarium lateritium*	leaf	MN833915	100
Nd-251	Pleosporales	*Paraconiothyrium archidendri*	leaf	OQ835627	100
Nd-257	Botryosphaeriales	*Neofusicoccum nonquaesitum*	leaf	MT587498	100
Nd-258	Pleosporales	*Alternaria consortialis*	leaf	KU726973	100
Nd-259	Eurotiales	*Penicillium crustosum*	leaf	MT582770	100
Nd-267	Diaporthales	*Coryneum ilicis*	leaf	NR171088	92.77
Nd-269	Diaporthales	*Diaporthe araucanorum*	stem	MN509710	100
Nd-273	Magnaporthales	*Pyricularia caricis*	stem	MK432729	99.38
Nd-274	Diaporthales	*Diaporthe araucanorum*	stem	MN509710	99.55
Nd-275	Diaporthales	*Coryneum modonium*	stem	MH674331	94.37
Nd-276	Eurotiales	*Penicillium crustosum*	stem	MT582770	100
Nd-278	Polyporales	*Phanerodontia magnoliae*	leaf	MK607594	100
Nd-279	Helotiales	*Hymenotorrendiella* sp.	leaf	OR565296	96.59
No-33	Diaporthales	*Diaporthe cynaroidis*	stem	ON193770	100
No-101	Hypocreales	*Clonostachys rosea*	stem	KP269006	100
No-129	Pleosporales	*Stemphylium globuliferum*	stem	OQ148588	99
No-137	Diaporthales	*Coryneum umbonatum*	stem	MH859114	92.59
No-142	Botryosphaeriales	*Neofusicoccum nonquaesitum*	leaf	MH864187	100
No-145	Sordariales	*Chaetomiun rectangulare*	leaf	OQ108372	99.82

Na: *N. alpina*; Nd: *N. dombeyi*; No: *N. oblicua*.

**Table 2:** Antibacterial activities of EF isolated from *Nothofagus* species.

Isolate	Species	Bc	Bs	Ea	Ec	Kp	Lm	Sal	Sa	Se	Sa *
Na-70	*F. petroliphilum* *	+++	+++	−	−	−	−	−	+++	+++	−
Na-71	*Pezicula* sp. *	−	−	−	−	−	−	−	−	−	−
Na-72	*Coryneum* sp. *	+++	+++	−	−	−	+++	−	+++	−	−
Na-78	*P. cinnamomea* *	+++	+++	−	++	++	+++	++	+++	+++	+++
Nd-5	*Trichoderma* sp. *	+++	++	+++	−	−	++	−	+++	−	++
Nd-9	*T. longibrachiatum* *	+++	−	++	−	++	−	++	−	+++	−
Nd-34	*C. rosea*	++	++	++	−	−	−	−	++	++	−
Nd-106	*P. chrysogenum*	−	−	−	−	−	−	−	−	−	−
Nd-150	*D. rudis*	−	−	−	−	−	−	−	−	−	−
Nd-151	*Alternaria alternata*	−	−	−	−	−	−	−	−	−	−
Nd-152	*P. cinnamomea*	−	−	−	−	++	−	−	−	−	−
Nd-250	*F. tricinctum* *	+++	−	+++	−	−	+++	++	+++	−	−
Nd-251	*P. archidendri*	−	−	−	−	−	−	−	−	−	−
Nd-257	*N. nonquaesitum*	−	−	−	−	−	−	−	−	−	−
Nd-258	*Alternaria* sp. *	++	−	++	−	−	++	−	−	++	−
Nd-259	*P. crustosum*	−	−	++	−	−	++	−	−	−	−
Nd-267	*C. ilicis*	−	−	−	−	−	−	−	−	−	−
Nd-269	*D. araucanorum*	−	++	−	−	++	−	−	++	−	−
Nd-273	*Pyricularia caricis*	−	−	−	−	−	−	−	−	−	−
Nd-274	*D. araucanorum*	−	−	−	++	−	−	++	−	−	−
Nd-275	*C. modonium*	−	−	−	−	−	−	−	−	−	−
Nd-276	*P. crustosum*	−	−	−	−	−	−	−	−	−	−
Nd-278	*P. magnoliae*	−	−	−	−	−	−	−	−	−	−
Nd-279	*Hymenotorrendiella* sp.	−	−	−	−	−	−	−	−	−	−
No-33	*D. cynaroidis*	++	++	−	−	−	−	−	−	−	−
No-101	*C. rosea* *	++	++	−	++	−	−	++	+++	−	−
No-129	*Stemphyllum* sp. *	++	++	−	−	−	−	−	+++	++	++
No-137	*C. umbonatum*	−	−	−	−	−	−	−	−	−	−
No-142	*N. nonquaesitum*	−	−	−	−	−	−	−	−	−	−
No-145	*C. rectangulare*	−	++	−	−	−	−	++	−	−	−

Bc: *B. cereus;* Bs: *B. subtilis*; Ea: *E. aerogenes;* Ec: *E. coli*; Kp: *K. pneumoniae;* Lm: *L. monocytogenes*; Sal: *S. enteriditis*; Sa: *S. aureus*; Se: *S. epidermidis*; Sa *: *S. aureus* MRSA. The activities were classified according to the diameter of the inhibition halo, expressed in millimeters (mm): +++ (active) = 15 mm or more, ++ (moderate) = 10–15 mm, − = no inhibition halo. * Isolate identified using phylogenetic trees.

**Table 3:** Antifungal activities of EF isolated from *Nothofagus* species.

Isolate	Species	Aa	An	Bc	Cg	Fo	Pe	Rs
Na-70	*F. petroliphilum* *	−	−	−	−	−	−	−
Na-71	*Pezicula* sp. *	+++	+++	−	+++	−	−	++
Na-72	*Coryneum* sp. *	+++	+++	−	+++	−	−	++
Na-78	*P. cinnamomea* *	+++	+++	+++	+++	+++	+++	+++
Nd-5	*Trichoderma* sp. *	++	−	−	−	−	−	−
Nd-9	*T. longibrachiatum* *	−	−	−	−	−	−	−
Nd-34	*C. rosea*	−	−	−	−	−	−	−
Nd-106	*P. chrysogenum*	−	−	−	−	−	−	−
Nd-150	*D. rudis*	−	−	−	−	−	−	−
Nd-151	*Alternaria alternata*	−	−	−	−	−	−	−
Nd-152	*P. cinnamomea*	−	−	−	−	−	−	−
Nd-250	*F. tricinctum* *	−	−	−	−	−	−	−
Nd-251	*P. archidendri*	−	−	−	−	−	−	−
Nd-257	*N. nonquaesitum*	−	−	++	−	−	−	−
Nd-258	*Alternata* sp. *	−	+++	−	+++	−	−	−
Nd-259	*P. crustosum*	−	−	−	−	++	−	−
Nd-267	*C. ilicis*	−	−	++	−	−	−	−
Nd-269	*D. araucanorum*	−	−	−	−	−	++	−
Nd-273	*Pyricularia caricis*	−	−	−	++	−	−	−
Nd-274	*D. araucanorum*	−	−	−	−	−	−	−
Nd-275	*C. modonium*	−	−	−	−	−	++	−
Nd-276	*P. crustosum*	−	−	−	−	−	++	−
Nd-278	*P. magnoliae*	−	−	−	−	−	−	−
Nd-279	*Hymenotorrendiella* sp.	−	−	−	−	−	−	−
No-33	*D. cynaroidis*	−	−	−	−	−	−	−
No-101	*C. rosea* *	+++	++	−	++	++	+++	+++
No-129	*Stemphyllum* sp. *	−	−	−	−	−	−	−
No-137	*C. umbonatum*	−	−	−	−	−	−	−
No-142	*N. nonquaesitum*	−	++	−	−	−	++	−
No-145	*C. rectangulare*	−	−	−	−	−	−	−

Aa: *A. alternata*; An: *A. niger*; Bc: *B. cinerea*; Cg: *C. gloesporioides*; Fo: *F*. oxysporum; Pe: *P. expansum*; Rs: *R. stolonifer*. The activities were classified according to the diameter of the inhibition halo, expressed in millimeters (mm) +++ (active) = 15 mm or more, ++ (moderate) = 10–15 mm, − = no inhibition halo. * Isolate identified using phylogenetic trees.

**Table 4:** Determination of MIC of the EtOAc extracts with antibacterial activity.

Isolate	Bc	Bs	Ea	Lm	Sa	Se	Sa *
*Trichoderma* sp.-5	500 ^a^	-	500 ^a^	-	500 ^a^	nt	-
*T. longibrachiatum*-9	500 ^a^	nt	-	nt	nt	nt	nt
*F. petroliphilum*-70	500 ^a^	500 ^a^	nt	nt	nt	nt	500 ^a^
*Pezicula* sp.-71	nt	nt	nt	nt	nt	nt	nt
*Coryneum* sp.-72	50 ^a^ 200 ^b^	50 ^a^ 200 ^b^	nt	50 ^a^ 200 ^b^	50 ^a^ 100 ^b^	nt	nt
*P. cinnamomea*-78	50 ^a^ 100 ^b^	50 ^a^ 100 ^b^	nt	500 ^a^	50 ^a^ 100 ^b^	500 ^a^	500 ^a^
*C. rosea*-101	-	-	nt	nt	200 ^a^ 500 ^b^	nt	nt
*Stemphylum* sp.-129	-	-	nt	nt	200 ^a^ 500 ^b^	-	nt
*F. tricinctum*-250	500 ^a^	nt	50 ^a^ 100 ^b^	50 ^a^ 100 ^b^	500 ^a^	nt	nt
*Alternaria* sp.-258	nt	nt	nt	nt	nt	nt	nt

-: No effect at 500 µg/mL; ^a^: bacteriostatic effect; ^b^: bactericidal effect; nt: no tested. Bc: *B. cereus;* Bs: *B. subtilis*; Ea: *E. aerogenes;* Lm: *L. monocytogenes*; Sa: *S. aureus*; Se: *S. epidermidis*; Sa *: *S. aureus* MRSA.

**Table 5:** Determination of MIC of the EtOAc extracts with antifungal activity.

Isolate	Aa	An	Bc	Cg	Fo	Pe	Rs
*Trichoderma* sp.-5	500 ^a^	nt	nt	nt	nt	nt	nt
*T. longibrachiatum*-9	nt	nt	nt	nt	nt	nt	nt
*F. petroliphilum*-70	nt	nt	nt	nt	nt	nt	nt
*Pezicula* sp.-71	100 ^a^ 500 ^b^	100 ^a^ 500 ^b^	nt	100 ^a^ 500 ^b^	nt	nt	500 ^a^
*Coryneum* sp.-72	100 ^a^ 500 ^b^	100 ^a^ 500 ^b^	nt	100 ^a^ 500 ^b^	nt	nt	500 ^a^
*P. cinnamomea*-78	100 ^a^ 500 ^b^	100 ^a^ 500 ^b^	10 ^a^ 50 ^b^	10 ^a^ 50 ^b^	200 ^a^ 500 ^b^	200 ^a^ 500 ^b^	200 ^a^ 500 ^b^
*C. rosea*-101	100 ^a^ 500 ^b^	-	nt	500 ^a^	nt	100 ^a^ 500 ^b^	100 ^a^ 500 ^b^
*Stemphylum* sp.-129	nt	nt	nt	nt	nt	nt	nt
*F. tricinctum*-250	nt	nt	nt	nt	nt	nt	nt
*Alternaria* sp.-258	nt	100 ^a^ 500 ^b^	nt	100 ^a^ 500 ^b^	nt	nt	nt

-: No effect at 500 µg/mL; ^a^: fungistatic effect; ^b^: fungicidal effect; nt: no tested; Aa: *A. alternata*; An: *A. niger*; Bc: *B. cinerea*; Cg: *C. gloesporioides*; Fo: *F*. oxysporum; Pe: *P. expansum*; Rs: *R. stolonifer*.

**Table 6:** Effects of EtOAc extract produced by *P. cinnamomea*-78 on mycelial growth and spore germination (%).

	*A. alternata*		*A. niger*		*B. cinerea*		*C. gloesporioides*	
Treatments (µg/mL)	MGI	SGI	MGI	SGI	MGI	SGI	MGI	SGI (%)
10	8 ^a^	52 ^a^	5 ^a^	42 ^a^	15 ^a^	54 ^a^	5 ^a^	35 ^a^
100	11 ^b^	67 ^b^	7 ^b^	54 ^b^	24 ^b^	71 ^b^	9 ^b^	58 ^b^
200	15 ^b^	72 ^b^	10 ^b^	67 ^c^	34 ^b^	88 ^c^	20 ^b^	77 ^c^
500	29 ^b^	77 ^b^	18 ^c^	71 ^c^	42 ^b^	100 ^d^	42 ^c^	100 ^d^
1000	34 ^b^	94 ^c^	32 ^d^	77 ^d^	60 ^c^	100 ^d^	57 ^d^	100 ^d^
2000	42 ^c^	97 ^c^	49 ^e^	91 ^e^	77 ^d^	100 ^d^	64 ^e^	100 ^d^

MGI: Mycelial Growth Inhibition; SGI: Spore Germination Inhibition; Values in a column with different letters are significantly different according to the Tukey test (*p* = 0.05).

**Table 7:** Effects of EtOAc extract produced by *Coryneum* sp.-72 on mycelial growth and spore germination (%).

	*A. alternata*		*A. niger*		*C. gloesporioides*		*R. stolonifer*	
Treatments µg/mL	MGI	SGI	MGI	SGI	MGI	SGI	MGI	SGI
10	4 ^a^	34 ^a^	7 ^a^	35 ^a^	6 ^a^	15 ^a^	3 ^a^	8 ^a^
100	10 ^b^	58 ^b^	8 ^a^	54 ^b^	18 ^b^	52 ^b^	4 ^a^	12 ^b^
200	17 ^c^	77 ^c^	9 ^a^	63 ^c^	24 ^b^	68 ^b^	10 ^b^	23 ^c^
500	29 ^d^	88 ^d^	17 ^b^	72 ^c^	33 ^c^	74 ^b^	15 ^b^	43 ^d^
1000	32 ^d^	92 ^d^	20 ^b^	77 ^c^	39 ^c^	87 ^c^	18 ^b^	60 ^d^
2000	45 ^e^	97 ^e^	33 ^c^	86 ^d^	43 ^d^	92 ^d^	25 ^c^	68 ^d^

MGI: Mycelial Growth Inhibition; SGI: Spore Germination Inhibition; Values in a column with different letters are significantly different according to the Tukey test (*p* = 0.05).

**Table 8:** Characterization of the spots and Retention factors (Rf).

	*Coryneum sp.-72*		*P. cinnamomea-78*	
Spot	254/365 nm	Rf	254/365 nm	Rf
1	black/orange	0.39	black/light blue	0.07
2	black/Nd	0.81	black/light green	0.54
3	-	-	black/light green	0.61
4	-	-	black/light green	0.65
5	-	-	black/purple	0.70
6	-	-	black/light green	0.74
7	-	-	black/light blue	0.81
8	-	-	black/brown	0.95

Nd: not detected.

## Data Availability

The data presented in the study are available on request from the corresponding author.

## Supplementary Materials

The following supporting information can be downloaded at: https://www.mdpi.com/article/10.3390/jof12010077/s1.
