# Supplementary material for: Antimicrobial Activity of Submerged Cultures of Endophytic Fungi Isolated from Three Chilean Nothofagus Species
Source: J Fungi (Basel). 2026 Jan 21;12(1):77. doi: 10.3390/jof12010077 (PMC12842994; doi:10.3390/jof12010077)
Supplement: Supplementary file 1 [file jof-12-00077-s001.zip › jof-3999516-supplementary.pdf]

Supplementary information

Antimicrobial Activity of Submerged Cultures of Endophytic Fungi Isolated from Three Chilean *Nothofagus* Species

Héctor Valenzuela [1], Daniella Aqueveque-Jara [1], Mauricio Sanz [1], Margarita Ocampo [1], Karem Henríquez-Aedo [2], Mario Aranda [3] and Pedro Aqueveque[1*]

[1] Laboratory of Microbiology and Mycology Applied, Department of Agroindustries, Faculty of Agricultural Engineering, University of Concepcion, Chillan, Chile; hvalenzuela2016@udec.cl (H.V.); daqueveque2017@udec.cl (D.A.J.); msanz2016@udec.cl (M.S.); mocampo@udec.cl (M.O.).

[2] Laboratory of Biotechnology and Food Genetics, Basic Sciences Department, Faculty of Sciences, University of Bio-Bio, Chillan, Chile; kahenriquez@ubiobio.cl (K.H.A.)

[3] Laboratory of Food & Drug Research, Department of Pharmacy, Faculty of Chemistry and Pharmacy, Pontifical Catholic University of Chile, Santiago, Chile; mario.aranda@uc.cl (M.A.)

* Correspondence: pedroaqueveque@udec.cl

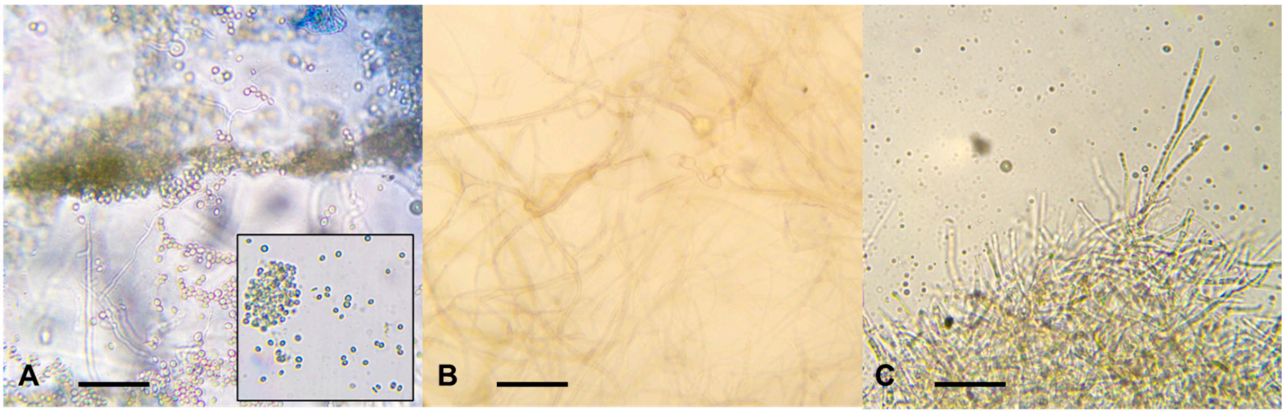

Figure S1. Micromorphological characteristics of EF from *Nothofagus.* A. *Trichoderma* sp.-5, and spores, B. *Coryneum* sp.-72, C. *P. cinnamomea*-78, Scale bars: (A-C) = 50 µm.

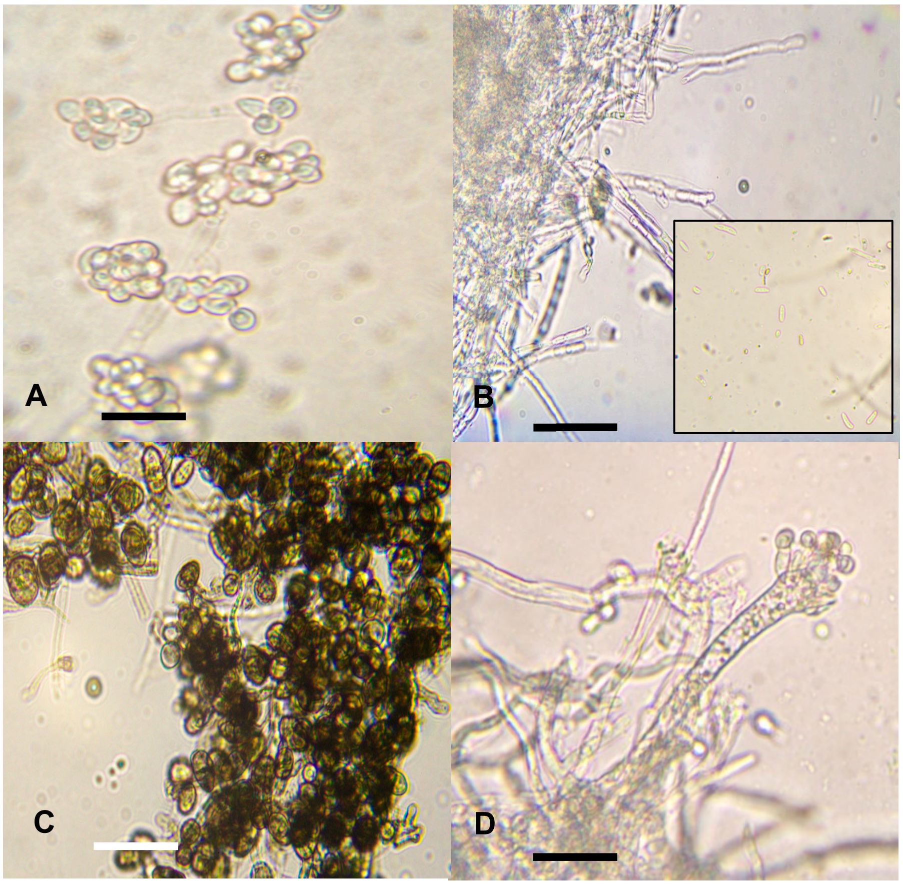

Figure S2. Micromorphological characteristics of EF from *Nothofagus.* A. *C. rosea*-101, B. *F. tricinctum*-250, C. *Alternaria* sp.-258, D. *P. crustosum*-259. Scale bars: (A-D) = 50 µm.

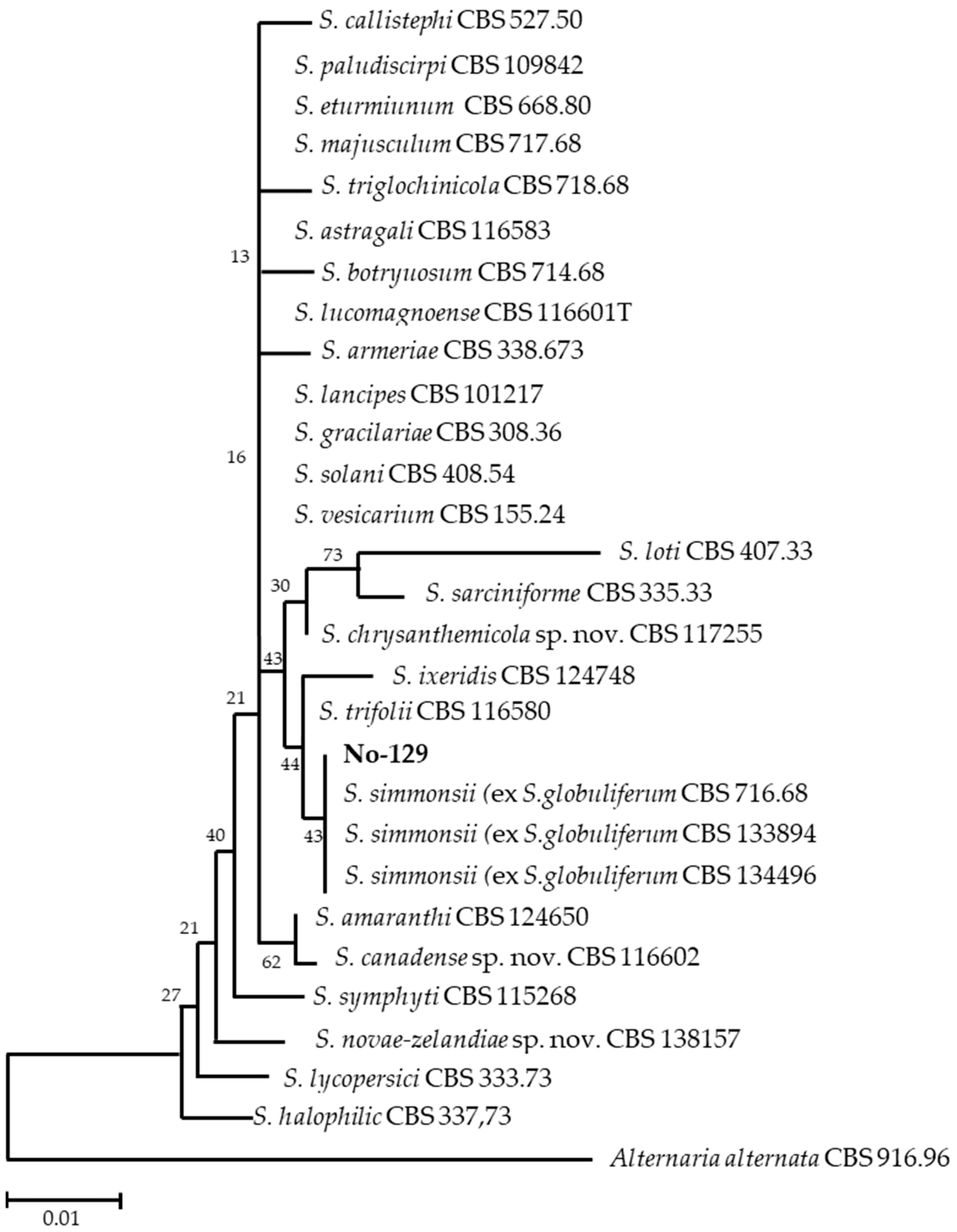

Figure S3. *Stemphylium.* Neighbor-joining tree based on ITS sequences of isolates No-129 isolated from *Nothofagus* genus. The tree was rooted in *Alternaria alternata* (outgroup). Numbers labeled at each node indicate bootstrap value (%) from 500 replicated.

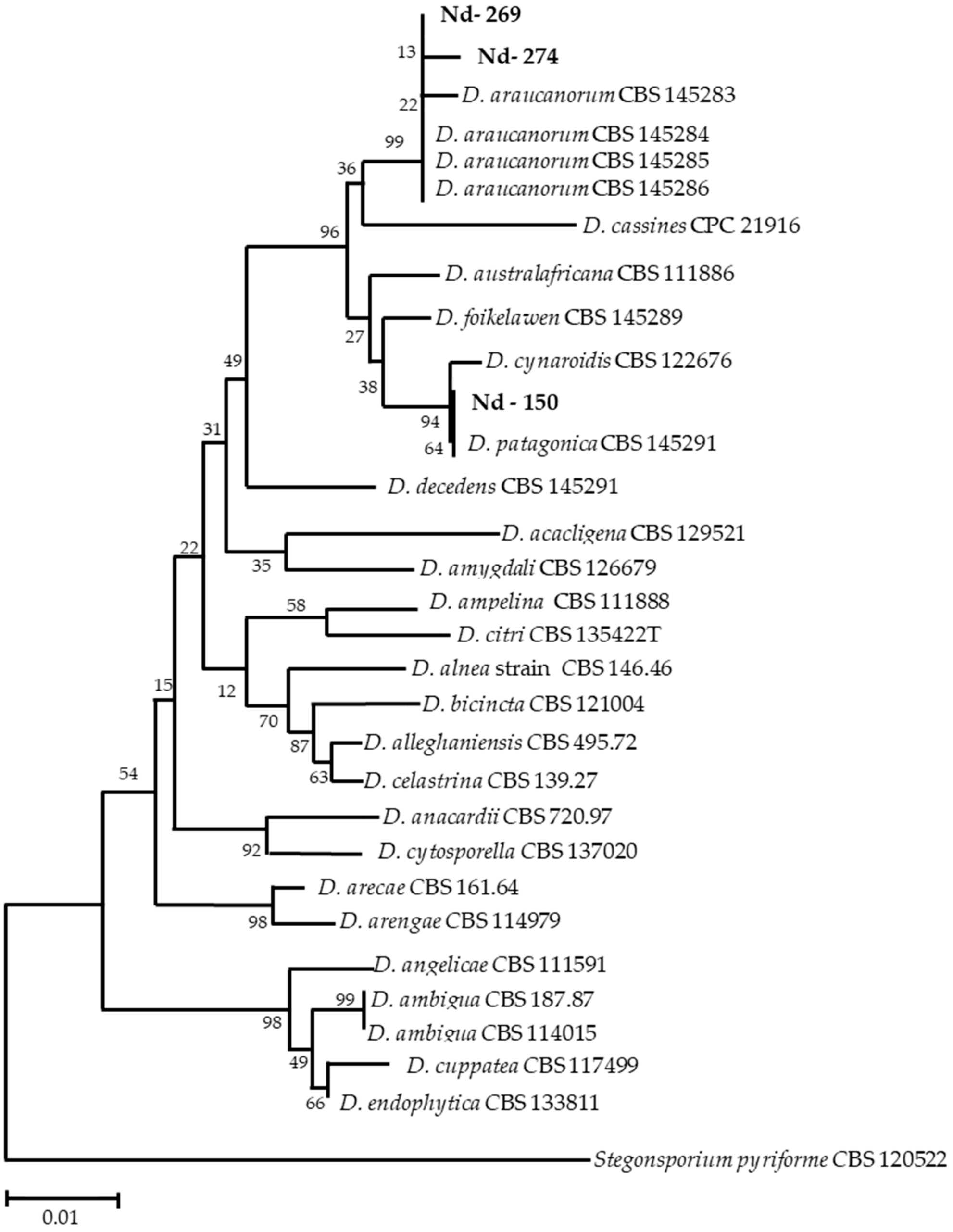

Figure S4. *Diaporthe.* Neighbor-joining tree based on ITS sequences of isolates Nd-269 and Nd-274 isolated from *Nothofagus* genus. The tree was rooted in *Stegonsporium pyriforme* (outgroup). Numbers labeled at each node indicate bootstrap value (%) from 500 replicated.

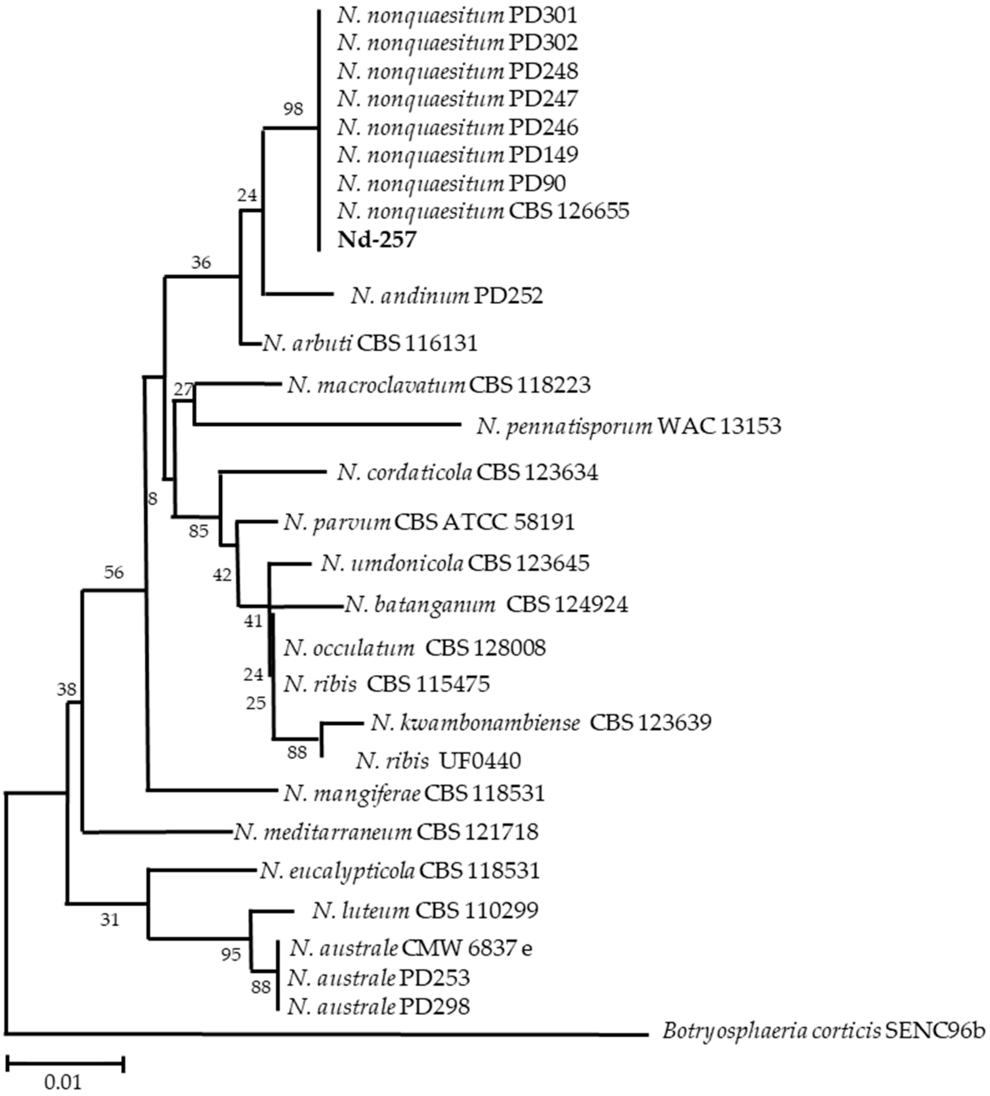

Figure S5. *Neofusicoccum.* Neighbor-joining tree based on ITS sequences of isolates Nd-257 isolated from *Nothofagus* genus. The tree was rooted in *Botryosphaeria corticis* (outgroup). Numbers labeled at each node indicate bootstrap value (%) from 500 replicated.

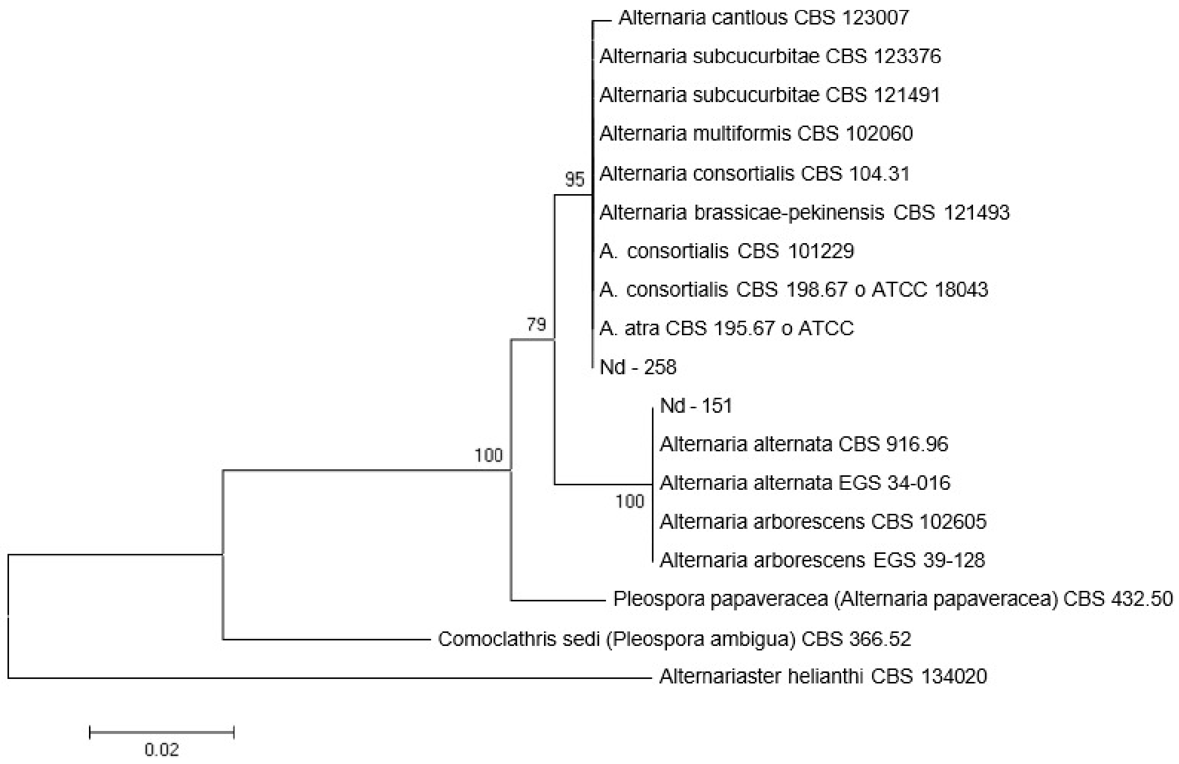

Figure S6. *Alternaria.* Neighbor-joining tree based on ITS sequences of isolates Nd-151 and Nd-258 isolated from *Nothofagus* genus. The tree was rooted in *Alternariaster helianthi* (outgroup). Numbers labeled at each node indicate bootstrap value (%) from 500 replicated.

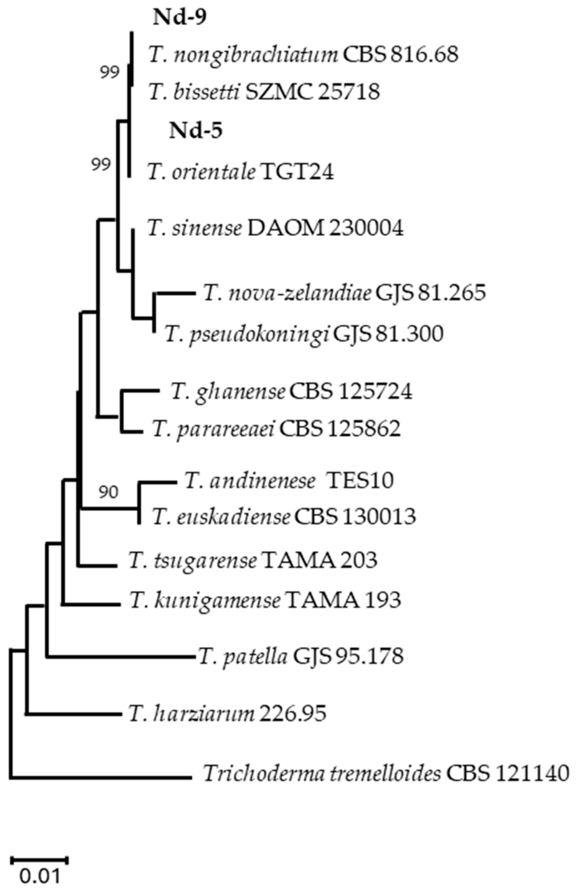

Figure S7. *Trichoderma.* Neighbor-joining tree based on ITS sequences of isolates Nd-5 and Nd-9 isolated from *Nothofagus* genus. The tree was rooted in *Trichoderma tremelloides* (outgroup). Numbers labeled at each node indicate bootstrap value (%) from 500 replicated.
